# Targeting connexin 43 with α–connexin carboxyl-terminal (ACT1) peptide enhances the activity of the targeted inhibitors, tamoxifen and lapatinib, in breast cancer: clinical implication for ACT1

**DOI:** 10.1186/s12885-015-1229-6

**Published:** 2015-04-03

**Authors:** Christina L Grek, Joshua Matthew Rhett, Jaclynn S Bruce, Melissa A Abt, Gautam S Ghatnekar, Elizabeth S Yeh

**Affiliations:** 1 FirstString Research, Inc., 300 W. Coleman Blvd., Suite 203, Mount Pleasant, SC USA; 2 Department of Surgery, Division of General Surgery, Medical University of South Carolina, Charleston, SC USA; 3 Department of Cell and Molecular Pharmacology and Experimental Therapeutics, Medical University of South Carolina, 173 Ashley Ave, BSB358, MSC509, Charleston, SC 29425 USA

**Keywords:** Connexin43, ACT1 peptide, Cancer therapeutic, Gap junction intercellular communication, Lapatinib, Tamoxifen, Breast cancer

## Abstract

**Background:**

Treatment failure is a critical issue in breast cancer and identifying useful interventions that optimize current cancer therapies remains a critical unmet need. Expression and functional studies have identified connexins (Cxs), a family of gap junction proteins, as potential tumor suppressors. Studies suggest that Cx43 has a role in breast cancer cell proliferation, differentiation, and migration. Although pan-gap junction drugs are available, the lack of specificity of these agents increases the opportunity for off target effects. Consequently, a therapeutic agent that specifically modulates Cx43 would be beneficial and has not been tested in breast cancer. In this study, we now test an agent that specifically targets Cx43, called ACT1, in breast cancer.

**Methods:**

We evaluated whether direct modulation of Cx43 using a Cx43-directed therapeutic peptide, called ACT1, enhances Cx43 gap junctional activity in breast cancer cells, impairs breast cancer cell proliferation or survival, and enhances the activity of the targeted inhibitors tamoxifen and lapatinib.

**Results:**

Our results show that therapeutic modulation of Cx43 by ACT1 maintains Cx43 at gap junction sites between cell-cell membrane borders of breast cancer cells and augments gap junction activity in functional assays. The increase in Cx43 gap junctional activity achieved by ACT1 treatment impairs proliferation or survival of breast cancer cells but ACT1 has no effect on non-transformed MCF10A cells. Furthermore, treating ER+ breast cancer cells with a combination of ACT1 and tamoxifen or HER2+ breast cancer cells with ACT1 and lapatinib augments the activity of these targeted inhibitors.

**Conclusions:**

Based on our findings, we conclude that modulation of Cx43 activity in breast cancer can be effectively achieved with the agent ACT1 to sustain Cx43-mediated gap junctional activity resulting in impaired malignant progression and enhanced activity of lapatinib and tamoxifen, implicating ACT1 as part of a combination regimen in breast cancer.

**Electronic supplementary material:**

The online version of this article (doi:10.1186/s12885-015-1229-6) contains supplementary material, which is available to authorized users.

## Background

Gap junctions are specialized membrane channels that facilitate intercellular communication through the exchange of ions, second messengers, and small metabolites (generally < 1 kDa in size) between neighboring cells and enable electrical propagation in excitable tissues [1]. The core protein components of gap junction channels are connexins (Cxs), tetraspan transmembrane proteins containing two extracellular loops, a cytoplasmic loop, and cytoplasmic N-terminal and C-terminal domains. Six connexins oligomerize to form cored transmembrane hemichannels that couple with hemichannels on neighboring cells to form intercellular channels. In turn, these intercellular channels aggregate to form gap junctions [2,3]. The human genome encodes for 21 connexin genes, each with a tissue and cell-type specific expression pattern.

Gap junctions have a widely recognized role in tumorigenesis and the progression of metastatic disease where research has highlighted individual connexin proteins as potential tumor suppressors that regulate tumor cell proliferation and tumor growth in vivo [4]. Several studies have implicated Cx43 in mammary gland development [5-10] and investigated Cx43 expression in breast cancer cells or human breast cancer tissues [5,11-18]. These studies propose that Cx43 has a role in breast cancer cell proliferation, differentiation, and migration. Additionally, studies that examine human breast cancer tissue indicate that Cx43 expression patterns fluctuate with cancer stage [14-16,19]. These studies also suggest that decreased Cx43 localization to gap junctions can mediate disease severity independently of Cx43 expression levels and thus loss of Cx43 at gap junctions could act as a biomarker of malignancy [14-17,19]. Consequently, maintaining Cx43 function at gap junctions, thereby preserving gap junction intercellular communication, has the potential to attenuate malignant transformation and metastatic progression [14,16,17].

Unfortunately, the development and evaluation of therapeutic interventions aimed at targeting Cx43 in breast cancers is complicated due to evidence that connexins may have differential and dynamic roles during tumor cell dissemination. The loss of gap junction intercellular communication corresponds with the initial stages of malignant phenotype progression in neoplastic mammary tissue and may be related to changes in cell-cell adhesion. Consistent with this concept, it has been reported that the loss of gap junctions contributes toward allowing cells to physically detach leading to invasion and metastatic disease progression [4,20-22]. Conversely, reports indicate that Cx43 is upregulated in established breast cancer metastatic lesions, suggesting that connexins may play roles in late metastatic steps involving extravasation and tissue colonization [11,15,23,24]. Additionally, Cx43 expression has been shown to shift to stromal compartments during cancer progression, suggesting that Cx43 may be regulating invasion and metastasis through interactions between epithelial tumor cells and the stroma [25]. It is important to acknowledge that conflicting data likely represent differences in experimental approaches, the cellular heterogeneity of tumors, the overlapping roles of other connexin family members, and the complexity of the metastatic process.

It is likely that channel-independent functions also contribute to Cx43’s role in breast cancer. Because it is possible that differential activities for Cx43 are relevant in breast cancer, a method for examining endogenous Cx43 activity at gap junctions would be instrumental in clarifying the role of Cx43 in breast cancer and the therapeutic potential of targeting this molecule. However, studies examining the role of Cx43 in mammary/breast cells have used either drugs aimed at the global inhibition of gap junction activity that are not specific for Cx43, or overexpression constructs, which alter Cx43 mRNA and protein expression levels therefore adding an additional variable to experimental interpretation [6,26-32].

In order to specifically examine endogenous Cx43 activity at gap junctions without altering expression levels, we have employed a unique 25-amino acid length peptide drug (ACT1) that mimics a cytoplasmic regulatory domain of Cx43. ACT1 redirects uncoupled Cx43 hemichannels into gap junctions, thereby reducing gap junction turnover and enhancing gap junction aggregation, without effecting Cx43 protein levels [33,34]. Consequently, ACT1 provides the desired characteristics of separating Cx43 expression levels from Cx43 function, in order to study the gap-junctional activity of Cx43 in breast cancer. Here, we present evidence demonstrating that ACT1-mediated augmentation of Cx43-composed gap junctions impairs proliferation or induces apoptosis in breast cancer cells but not in non-transformed mammary epithelial cells, confirming a tumor suppressive function for Cx43.

Furthermore, stabilizing junctional Cx43 and modulating gap junctional intercellular communication in cancer cells has been suggested to elicit a “bystander” effect, where increases in the number or size of gap junctions and enhanced gap junctional intercellular communication result in increased diffusion of cytotoxic agents, sensitization to chemotherapeutics, and an amplification of therapeutic response [4,35]. Supporting this assertion, re-expression of Cx43 in human cancer cells has been shown to increase cancer cell sensitivity to common chemotherapeutic agents [36,37]. Conversely, a recent study by Munoz et al. showed that Cx43 levels were increased in temozolomide-resistant Glioblastoma Multiforme cells implying that gap junction intercellular communication between the resistant cells due to the increase in Cx43 might contribute to the development of temozolomide resistance [38]. These conflicting results could be due to differences in experimental system (i.e. breast versus brain) or highlight non-junctional activities of Cx43 in Glioblastoma Multiforme, as we now provide evidence to show that modulation of Cx43 signaling with ACT1 effectively increases gap junctional intercellular communication in breast cancer cells, and enhances drug-induced cytotoxicity of the targeted therapies tamoxifen in the ER+ MCF7 breast cancer cell line and lapatinib in the HER2+ BT474 breast cancer cell line. Our results demonstrate that modulation of Cx43-based gap junctional activity and distribution by ACT1 impairs breast cancer cell proliferation or survival and highlights ACT1 as a potential therapeutic agent in the treatment of breast cancer.

## Methods

### Cell culture

MCF10A cells were maintained in DMEM (Hyclone) supplemented with FBS (Gibco), 10 ng/ml EGF, 5 μg/ml insulin, and 1 μg/ml hydrocortisone. MCF7 cells were maintained in DMEM (Hyclone) supplemented with FBS (Gibco). MDA MB 231 cells were maintained in Leibovitz supplemented with FBS (Gibco). BT474 cells were maintained in RPMI-1640 supplemented with FBS (Gibco). All media contained 2 mM glutamine (Thermo Scientific) and Penicillin/Streptomycin (Pen/Strep, Thermo Scientific) unless otherwise specified.

### Therapeutic peptides

α–connexin carboxyl-terminal (ACT1) peptide and its reverse sequence peptide (R-pep) were synthesized by the American Peptide Company (Sunnyvale, CA). The ACT1 peptide corresponds to a short sequence at the Cx43 C-terminus linked to an antennapedia internalization sequence (RQPKIWFPNRRKPWKKRPRPDDLEI). Antennapedia internalization peptide sequence is RQPKIWFPNRRKPWKK. R-pep sequence consists of the reverse sequence of ACT1 attached to an anntennapedia internalization sequence.

### Gap-FRAP

Cells were treated with either ACT-1 (200 μM), reverse peptide (R-pep; 200 μM), or Vehicle (Veh; H_2_O) for 6 hr. One-half-hour prior to the end of the two hour incubation cells were loaded with calcein-AM. At the end of the incubation period, cells were washed and imaging medium (phenol-free Optimem) containing 100 μM of respective treatments was added to the culture. Cells were imaged on a Leica SP5 Laser Scanning Confocal Microscope using the FRAP wizard. Briefly, the cytoplasm of a single cell in the imaging field was bleached with 10 frames of high intensity laser light using all laser lines. Fluorescence recovery was monitored with 488 nm excitation every 10 sec over one minute. For BT474 and MCF7, N = 3 per treatment. For MCF10A, N = 2 per treatment. 3 replicates per sample per treatment.

### Crystal violet stain analysis

Equal numbers of cells were plated and treated the following day with ACT1, reverse peptide (R-pep), or Vehicle (Veh; H_2_O) for 48 hr. When indicated, cells were also treated with DMSO (vehicle), tamoxifen (Sigma), or lapatinib (Santa Cruz) prior to analysis. Following drug treatments, cells were fixed in 4% paraformaldehyde and stained with crystal violet. Cells were subsequently washed in deionized H_2_O and crystal violet was extracted with methanol. A540 was read using Benchmark Plus plate reader (Biorad).

### Cell counting analysis

Equal numbers of cells were plated and treated the following day with ACT1, reverse peptide (R-pep), or Vehicle (Veh; H_2_O) for 48 hr. When indicated, cells were also treated with DMSO (vehicle) or tamoxifen citrate (Sigma) prior to analysis by trypan blue exclusion and cell counting, which was performed using a hemocytometer or the Cellometer Mini cell counter (Nexcelom).

### Mammosphere assay

Mammosphere assay was performed as previously reported [39]. Briefly, 500 BT474 cells per well were plated into low adhesion 96 well plates (BrandTech Scientific Inc.). After 3 days in culture, peptides and targeted inhibitors were added to wells. On day 7 of the experiment, mammosphere structures were quantitated. Mammosphere forming efficiency was calculated using the formula: (number of mammospheres per well/number of cells seeded per well) × 100, as previously described [39].

### Immunoblotting

Cells were lysed directly into SDS Sample Buffer containing β-mercaptoethanol. Primary antibodies used for western blotting are: anti-Cx43 (Sigma, C-terminal directed antibody), anti-LC3B (Cell Signaling), anti-pAKT (Santa Cruz), anti-pERK1/2 (Santa Cruz), anti-cleaved-PARP (Cell Signaling), anti-ZO1 (cell signaling), anti-Cx26 (Santa Cruz), anti-Cx46 (Santa Cruz), anti-cleaved-Caspase-3 (Cell Signaling), and anti-β-tubulin (Santa Cruz). Protein expression levels were evaluated using the Odyssey imaging system (LICOR).

### Statistical analysis

For the gap-FRAP data an ANOVA was performed comparing the values at each time point and a Bonferroni post-hoc analysis was used to determine significance for the MCF-7 data. In the case of the BT474s a Dunnett’s Multiple Comparison Test was used. For all other analyses, Student’s *T*-Test was used and represented as Standard Error of the Mean. For all experiments, N = number of plates or wells. For some experiments, such as gap-FRAP analysis, multiple replicates (3 per N) were analyzed within each plate.

### Ethics statement

No human subjects, data, or materials were used in this study.

## Results

### ACT1 stabilizes gap junction intercellular communication in breast cancer cells

**Figure 1:**
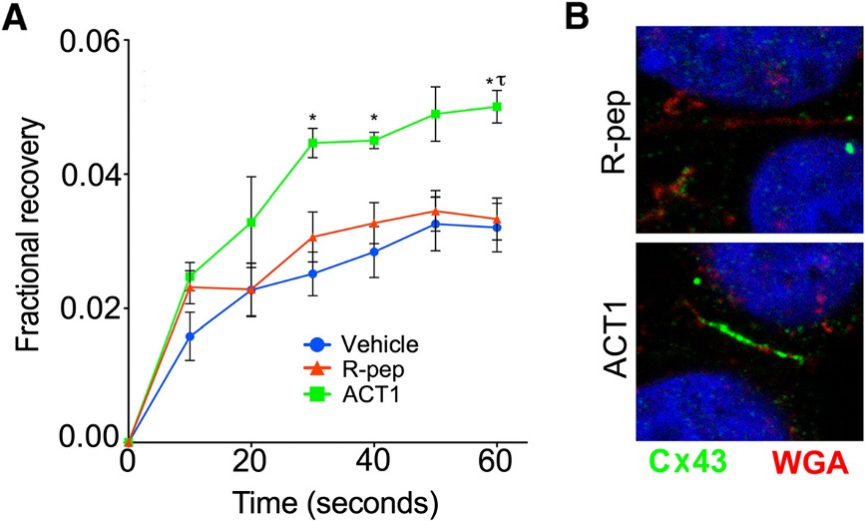
**Connexin 43 activity and expression after ACT1 treatment in MCF7 cells.** MCF7 cells were treated with vehicle, R-pep (200 μM), or ACT1 (200 μM) and assessed for **(A)** gap-FRAP. ANOVA with Bonferroni post-hoc analyses were used to determine statistical significance * = p < 0.05 vs Vehicle, *τ* = p < 0.05 vs R-Pep; ± SEM; n = 3 **(B)** Immunofluoresence staining and imaging of Cx43 (green) in MCF7 cells treated with R-pep or ACT1. Wheat germ agglutinin (WGA) in red was used to stain cell membranes.

Previously it was shown in non-breast cancer cell types that the therapeutic peptide mimetic ACT1, which targets Cx43 protein interaction and/or signaling, is able to increase gap junction hemichannel recruitment and size, thereby stabilizing gap junction intercellular communication without altering Cx43 levels [33,34]. To extend these observations and investigate the role of Cx43 gap junctions in breast cancer cells, we treated MCF7 cells with vehicle (water), a non-functional reverse peptide control (R-pep), or ACT1 and monitored for gap junctional activity using a Fluorescence Recovery After Photobleaching (FRAP)-based gap junction activity assay (gap-FRAP). Gap-FRAP measures the diffusion of a gap junction-permeable fluorescent dye (calcein-AM) allowing for quantitative measurement of gap junctional activity, since the rate of dye diffusion (i.e. recovery) is directly proportional to the amount of gap junctions that are coupled and active. We found that treatment of MCF7 cells with ACT1 increased dye diffusion measured by gap-FRAP more robustly than either vehicle or R-pep (Figure 1A), demonstrating that ACT1 is able to positively impact gap junction activity in MCF7 cells. These results were confirmed by immunofluorescence (IF) staining for Cx43 in R-pep and ACT1 treated MCF7 cells which showed increased localization of Cx43 protein at the membrane border between neighboring cells in ACT1 treated cell populations (Figure 1B). Also consistent with previous findings, we examined total Cx43 protein by immunoblotting extracts from MCF7 cells treated with water, R-pep, and ACT1 and saw no appreciable difference in overall Cx43 expression levels (Additional file 1: Figure S1).

It was previously shown that Cx43 inhibits autophagy and that this function of Cx43 is likely gap junction independent [36,40]. Therefore, we evaluated whether ACT1 treatment affects autophagy by examining LC3B processing in MCF7 cells after ACT1 treatment. We found no changes in LC3B modification between ACT1 treated cells and R-pep or water treated cells even in the presence of the autophagy inhibitor chloroquine (Additional file 1: Figure S2A). Additional studies indicate that AKT and MAPK, via ERK1/2, regulate Cx43 and its gap junction activity [41-43]. Consequently, we looked at AKT and ERK1/2 activity by monitoring phosphorylation of these molecules and found that ACT1 treatment did not alter AKT or ERK1/2 phosphorylation status (Additional file 1: Figure S2B). Taken together, our results demonstrate that ACT1 modulates the gap junctional activity of Cx43 by stabilizing endogenous Cx43 at membrane borders between cells.

### Targeting connexin 43 with ACT1 reduces proliferation of breast cancer cells

**Figure 2:**
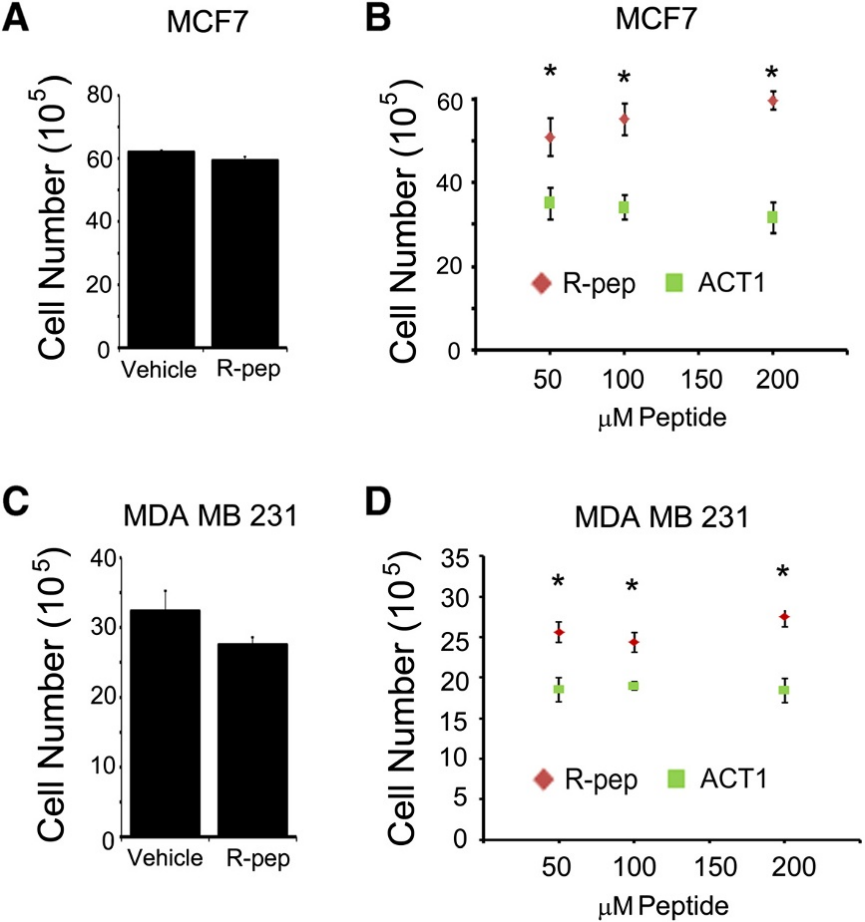
**Reduced proliferation of MCF7 and MDA MB 231 cells treated with ACT1. (A)** MCF7 cells were treated with vehicle or R-pep (200 μM) for 48 hours and assessed for total cell number. **(B)** MCF7 cells were treated for 48 hours with 50, 100, or 200 μM of R-pep or ACT1 and total cell number were compared at each drug concentration. **(C)** MDA MB 231 cells were treated with vehicle or R-pep (200 μM) for 48 hours and assessed for total cell number. **(D)** MDA MB 231 cells were treated for 48 hours with 50, 100, or 200 μM of R-pep or ACT1 and total cell number were compared at each drug concentration. Student’s *T*-test analysis was used to determine statistical significance. *p < 0.01; ± SEM; n = 8.

Previous studies have shown that overexpression of Cx43 decreases proliferation of breast cancer cells and this observation was attributed to increased localization of Cx43 to sites of gap junctions [31]. Given these observations and that Cx43 has been described as a tumor suppressor protein in breast cancer [44], we evaluated the effect of modulating Cx43 with ACT1 on breast cancer cell proliferation. MCF7 cells were treated with water in equal volume or increasing concentrations (50, 100, and 200 μM) of R-pep or ACT1 for 48 hr and evaluated for total cell number after treatment. To first demonstrate that the control R-pep did not have an appreciable effect on proliferation, we compared vehicle (water) treated cells and R-pep treated cells at the highest dose of peptide (200 μM). We found no difference in cell number after 48 hr of treatment with either of the control agents (Figure 2A). We next compared total cell number after treatment between R-pep and ACT1 treated MCF7 cells, and found that cell number was decreased in ACT1 (50, 100, and 200 μM) treated MCF7 cells compared to R-pep control at the same dosages (Figure 2B).

As the aforementioned study also evaluated MDA MB 231 cells in the context of Cx43 overexpression [31], we additionally looked at this cell type by the same analysis for proliferation. Similar to our findings in MCF7 cells, we found no effect of vehicle or R-pep on MDA MB 231 proliferation (Figure 2C) but saw a decrease in proliferation due to ACT1 (50, 100, and 200uM) treatment (Figure 2D). Taken together, these findings demonstrate that modulation of Cx43 using ACT1 impairs breast cancer cell proliferation.

### Modulation of connexin 43 activity with ACT1 does not alter proliferation of non-transformed MCF10A mammary epithelial cells

**Figure 3:**
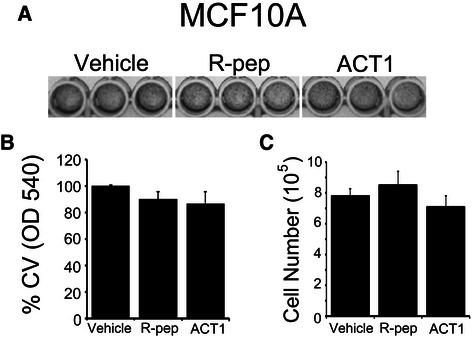
**ACT1 does not alter proliferation of non-transformed MCF10A mammary epithelial cells.** MCF10A cells were treated with vehicle, R-pep (200 μM), or ACT1 (200 μM) and assessed for **(A)** crystal violet staining density and **(B)** quantitated by OD540. **(C)** Cells were also assessed by cell counting. ± SEM; n = 6.

We next tested whether ACT1 treatment altered the proliferation of non-cancerous mammary epithelial cells. Therefore, we the evaluated proliferation of non-transformed MCF10A mammary epithelial cells treated with vehicle, R-pep, or ACT1. Equal numbers of cells were plated and treated with vehicle or 200 μM of R-pep or ACT1 for 48 hr. First, we evaluated cells by crystal violet (CV) staining, which revealed that an equivalent density of cells was present in each well, regardless of treatment (Figure 3A). We also quantitated the amount of CV stain in each well as a surrogate for total cell number and confirmed that there was no difference in the percent of total CV stain in each treatment group (Figure 3B). We additionally directly quantitated total cell number after performing the three treatments. Consistent with our observations in Figure 3A and B, no difference was observed in the total cell number between treatment groups (Figure 3C).

To further investigate why the non-transformed MCF10A cells behave differently compared to MCF7 and MDA MB 231 breast cancer cells in response to ACT1 treatment, we evaluated Cx43 intracellular distribution by IF and gap junction activity by gap-FRAP in MCF10A cells after R-pep and ACT1 treatment. We observed very sparse Cx43 staining in MCF10A cells that was primarily localized to the cytoplasm, rather than at membrane borders in both R-pep and ACT1 treated cells (Additional file 1: Figure S3A). We additionally observed little to no gap-FRAP activity in the MCF10A cells in two independent experiments, suggesting that these cells are gap junction deficient (Additional file 1: Figure S3B), which confirmed our observations by IF and also suggests that Cx43 could have differential function in non-transformed MCF10A cells. Together, these findings show that modulating Cx43 in non-cancerous MCF10A mammary epithelial cells does not alter proliferation, suggesting that ACT1-mediated modulation of gap junction activity toward reducing proliferation could be specific to breast cancer cells.

### ACT1 augments tamoxifen treatment of ER+ breast cancer cells

**Figure 4:**
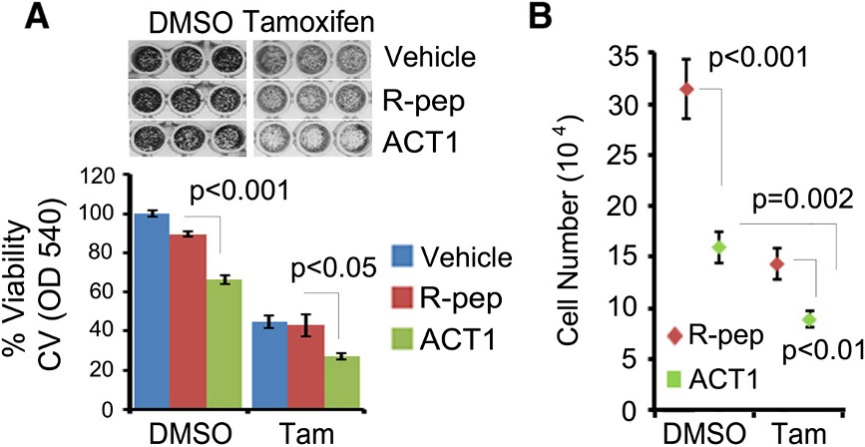
**ACT1 cooperates with tamoxifen to promote cytotoxicity in ER+ MCF7 cells.** MCF7 cells were treated with vehicle, R-pep (100 μM), or ACT1 (100 μM) in the presence of drug vehicle (DMSO) or tamoxifen citrate (10 μM) for 48 hours and assessed for **(A)** crystal violet staining density and quantitation by OD540. **(B)** R-pep and ACT1 treated cells were also assessed by cell counting after co-treatment with drug vehicle (DMSO) or tamoxifen citrate (10 μM) for 48 hours. Student’s *T*-test was used to assess statistical significance as indicated. ± SEM; n = 3 **(A)** n = 6 **(B)**.

In addition to being able to inhibit breast cancer cell proliferation on its own, as a gap junction stabilizer ACT1 has the added capacity to enhance intercellular coupling in a manner that promotes exchange of signals between cells, including cytotoxic signals that are initiated by cancer drug treatments [35]. This so-called “bystander” effect could be an added benefit of utilizing ACT1 for breast cancer treatment. To test this concept, we assessed dual treatment of MCF7 cells, which are estrogen receptor positive (ER+), with ACT1 and the ER antagonist tamoxifen. MCF7 cells were plated in equal number and subsequently treated with water, (100 μM) R-pep, or (100 μM) ACT1. Each individual group of treated cells (i.e. water, R-pep, or ACT1) was additionally treated with either a vehicle (DMSO) or 10 μM tamoxifen citrate. After 48 hr of dual treatment, cells were stained with CV and amount of CV incorporated into the cells was quantitated by OD540. Similar to our findings in Figure 2A and B, R-pep by itself had no effect on MCF7 cell quantity whereas ACT1 alone reduced the amount of MCF7 cells present (Figure 4A). When we evaluated vehicle, R-pep, or ACT1 cells that had also been treated with tamoxifen, we found that tamoxifen had a greater effect on reducing cell quantity in the ACT1 treatment group (Figure 4A), suggesting that ACT1 augments the activity of tamoxifen. We corroborated our results by performing additional experiments that evaluated total cell number, rather than using CV stain as a surrogate. Here, cells were treated with R-pep and ACT1 as indicated above but counted directly. These results showed again that dual ACT1 and tamoxifen treatment was more effective than ACT1 alone or R-pep plus tamoxifen dual treatment (Figure 4B).

### ACT1 enhances lapatinib activity on HER2+ breast cancer cells

**Figure 5:**
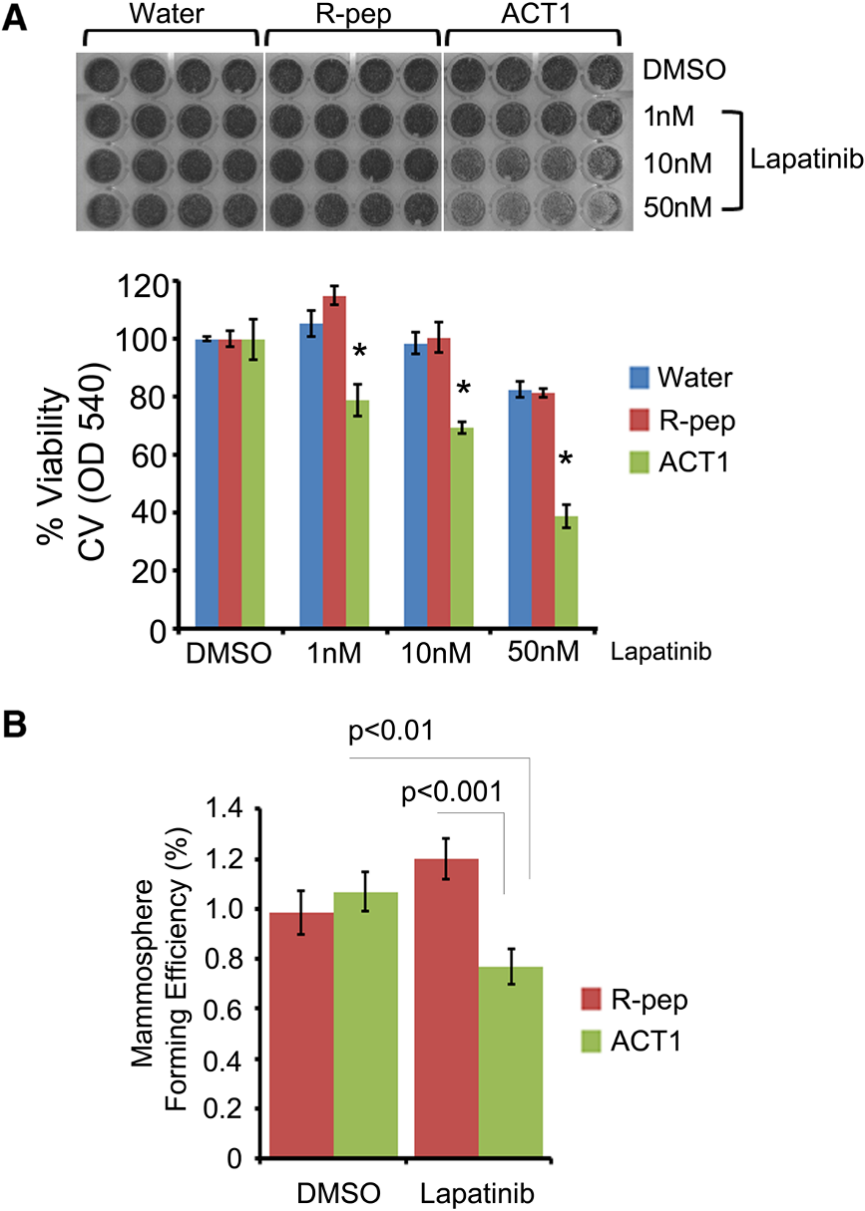
**ACT1 enhances the effectiveness of lapatinib in HER2+ BT474 cells. (A)** BT474 cells were treated with vehicle, R-pep (100 μM), or ACT1 (100 μM) in the presence of drug vehicle (DMSO) or lapatinib (1, 10, 50 nM) for 48 hours and assessed for crystal violet staining density and quantitation by OD540. Student’s *T*-test was used to assess statistical significance. *p ≤ 0.001 when ACT1 compared to either R-pep or vehicle control. **(B)** Combined treatment with ACT1 and lapatinib impairs BT474 mammosphere formation. BT474 cells were plated in ultra low adhesion 96-well plates for 72 hours, followed by treatment with R-pep (100 μM) or ACT1 (100 μM) in the presence of drug vehicle (DMSO) or lapatinib (50 nM) for 96 hours. At the end of the 7 day assays, wells were assessed for mammosphere number, which was used to calculate the mammosphere forming efficiency of the cells. Student’s *T*-test was used to assess statistical significance as indicated. ± SEM; n = 4 **(A)** n = 24 **(B)**.

To further test the ability of ACT1 to enhance targeted agents, we next evaluated Cx43 targeting in HER2+ BT474 breast cancer cells. Here, we first evaluated BT474 cells for ACT1 treatment-mediated effects on gap junction function by gap-FRAP and found that ACT1 treatment enhanced gap junction activity (Additional file 1: Figure S4A). Consistent with this observation, we also observed increased recruitment of Cx43 to the intercellular membrane border upon ACT1 treatment in IF-labeled fixed cell preparations (Additional file 1: Figure S4B). We next plated equal numbers of BT474 cells treating them with water, (100 μM) R-pep, or (100 μM) ACT1. Cells were then additionally treated with vehicle (DMSO) or lapatinib at 1 nM, 10 nM or 50 nM concentrations; levels of lapatinib that are generally lower than required to kill BT474 cells. While relatively little effect was seen in DMSO treated cells (Figure 5, first 3 columns), a significant reduction of cells was seen in the cells treated with the combination of ACT1 and lapatinib, with the most prominent difference at 50 nM of lapatinib treatment (Figure 5A). We next tested the effect of ACT1 and lapatinib on BT474 mammosphere formation. A four day drug treatment was performed after mammospheres were allowed to initially form for 3 days. Consistent with the above findings, we also observed a decrease in primary mammosphere number when BT474 cells were treated with the combination of ACT1 and lapatinib (Figure 5B). Our findings indicate that ACT1 and lapatinib negatively affected the ability of BT474 cells grow in 2D culture and to initiate mammosphere formation in 3D culture.

### ACT1-mediated changes in proliferation and apoptosis are differentially regulated in breast cancer cell lines

Previous studies suggest that downregulation of Cx43 increases proliferation and overexpression of Cx43 inhibits breast cancer cell proliferation [31]. However, ACT1 does not alter Cx43 expression levels (Additional file 1: Figure S1) [33,34]. To further pursue why proliferation was disrupted in MCF7 and MDA MB 231 cells but not MCF10A or BT474 cells, we next evaluated expression levels of p27 in MCF10A, MCF7, and BT474 cells in response to R-pep or ACT1 treatment. We chose to evaluate p27 because it was previously shown that p27 is upregulated by Cx43 in osteosarcoma and glioma cell lines resulting in cell cycle inhibition and thus, impaired proliferation [45-47]. To examine the effect of ACT1 on p27, we treated MCF10A, MCF7, and BT474 cells with R-pep or ACT1 and evaluated for p27 levels by immunoblotting. We found p27 levels were not altered by ACT1 (Additional file 1: Figure S5) suggesting that this is not the mechanism by which ACT1 inhibits proliferation. Subsequently, we pursued other possible reasons for this difference in proliferation.

**Figure 6:**
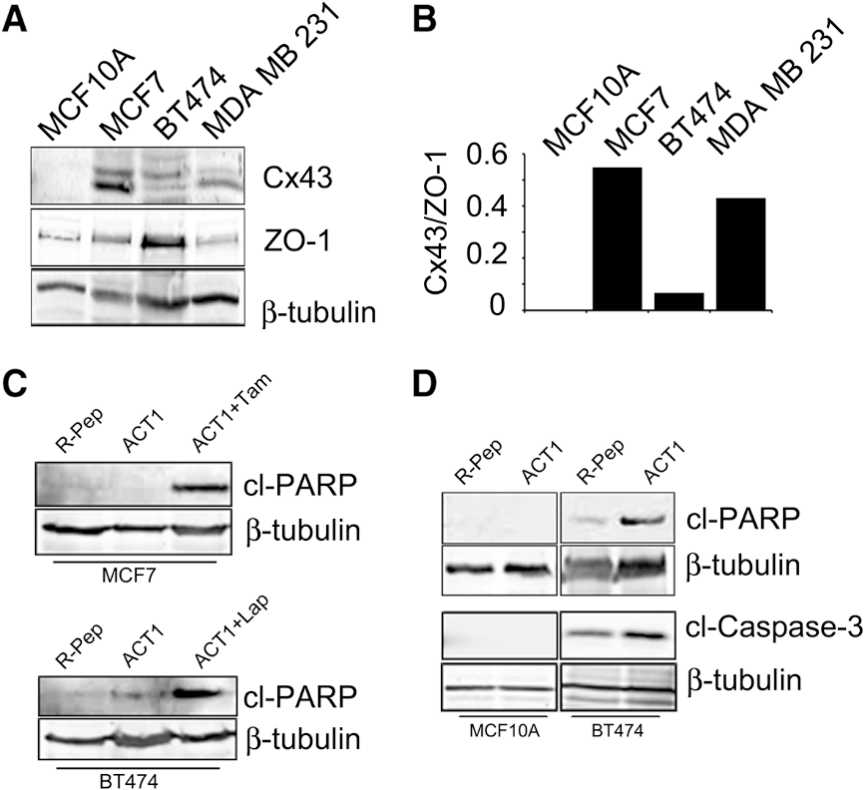
**Proliferation and apoptosis are differentially altered by ACT1 in MCF7 and BT474 breast cancer cell lines. (A)** Whole cell extracts isolated from MCF10A, MCF7, BT474, and MDA MB 231 cells were immunoblotted for Cx43 and ZO-1. **(B)** The ratio of Cx43 to ZO-1 expression was evaluated using the Odyssey imaging system. Cx43 and ZO-1 levels were normalized to β-tubulin expression. **(C)** MCF7 and BT474 cells were treated for 24 hours with R-pep (200 μM), ACT1 (200 μM), ACT1 plus Tamoxifen-MCF7, or ACT1 plus Lapatinib-BT474 and then analyzed for cleaved-PARP (cl-PARP) expression. **(D)** MCF10A and BT474 lysates treated with R-pep (200 μM) or ACT1(200 μM) were immunoblotted for cl-PARP and cleaved-Caspase-3 (cl-Caspase-3).

Because we observed differences in the effect of ACT1 on MCF10A versus MCF7 and MDA MB 231 cells as well as differential distribution of Cx43 by IF in MCF10A cells, this implied that Cx43 could be regulated differently in each of these cell lines. Earlier studies established that a mechanism of action for ACT1 is to disrupt Cx43 interaction with the tight junction protein, zonula occludens 1 (ZO-1) [33,34]. ACT1 mimics the C-terminal portion of Cx43 and in doing so competitively binds to ZO-1, which normally interacts with Cx43 and regulates Cx43 gap junction activity by sequestering Cx43 in a hemichannel state in the perinexus [33,48], thus limiting the rate of gap junction accretion. To evaluate the differences between the cell lines used in this study, we assessed Cx43 expression levels and the expression of its associated regulatory protein ZO-1. In addition to MCF7 cells and MDA MB 231 cells, which represent ER+ and TNBC cell lines respectively, we also evaluated BT474 HER2+ breast cancer cells. We found that Cx43 expression levels differed between cell types, with MCF10A and BT474 cells expressing low levels of Cx43 in comparison to MCF7 and MDA MB 231, which showed higher expression levels of Cx43 (Figure 6A). Additionally, we found significantly higher levels of ZO-1 in BT474 cells compared to the other three cell lines (Figure 6A) as well as a higher level of ZO-1 in comparison to Cx43 in both the MCF10A and BT474 cells. Consequently, if we calculated the ratio of Cx43 to ZO-1 expression in each of the cell lines, MCF10A and BT474 cells had very low Cx43 to ZO-1 ratio whereas MCF7 and MDA MB 231 cells had high levels of Cx43 compared to ZO-1 (Figure 6B). These findings suggest that ZO-1 could play a more significant role in regulating Cx43 in the BT474 cells and further implies that ACT1 activity on BT474 cells could be different from what we observe for other breast cancer cell types such as MCF7 cells.

To further investigate how ACT1 treatment differs between these two cell types we evaluated apoptosis. MCF7 and BT474 cells were treated with R-pep, ACT1, or a combination of ACT1 plus targeted inhibitors. We treated MCF7 cells with ACT1 plus tamoxifen whereas BT474 cells were treated with ACT1 plus lapatinib. Cells were exposed to these agents for 24 hours and then probed for cleaved-PARP as a marker for apoptosis. We found that ACT1 alone induced PARP cleavage in BT474 cells but had no effect on MCF7 cells (Figure 6C and D). The combination of ACT1 and lapatinib induced an even more robust response in the BT474 cells (Figure 6C, bottom panel). While no apoptosis was observed in MCF7 cells treated with ACT1 alone, the addition of tamoxifen was able to induce PARP cleavage suggesting that MCF7 cells may respond differently to ACT1 in terms of initiating cell death when compared to BT474 cells, which were responsive to ACT1. Our observations in Figure 6C also suggest that MCF7 cells undergo apoptosis mainly in response to tamoxifen. We confirmed our findings with ACT1 in the BT474 cell line by probing for a second apoptosis marker, cleaved-Caspase-3, which was also elevated in response to ACT1 treatment (Figure 6D). We also evaluated the effect of ACT1 on inducing cell death in MCF10A cells. No apoptosis was observed in MCF10A cells in response to ACT1 (Figure 6D). We did not evaluate cleaved-Caspase-3 in MCF7 cells because they do not express Caspase-3. Taken together, these observations indicate that changes in proliferation and apoptosis are differentially induced by ACT1 in BT474 cells compared to MCF7 cells. Furthermore, as a whole our findings show that combining ACT1 with the targeted agents, lapatinib and tamoxifen, will enhance the activity of these agents and argues for application of ACT1 as part of a combination regimen in breast cancer.

## Discussion

Our current findings demonstrate a role for the gap junction protein Cx43 in breast cancer growth and progression. Using a therapeutic agent, ACT1, that targets endogenous Cx43 by stabilizing its activity at gap junctions, we show that modulation of Cx43 signaling alters breast cancer cell proliferation in the MCF7 and MDA MB 231 cell lines. While these results are in agreement with a tumor suppressive role for Cx43 [44], our findings with the MDA MB 231 cell line is in disagreement with previous findings that used Cx43 overexpression to analyze proliferation of MDA MB 231 cells in 2D culture, which was not affected by Cx43 overexpression [31]. This difference in our findings could be attributed to the possibility that high levels of Cx43 due to experimental overexpression alters MDA MB 231 cells in a manner that is dissimilar to modulation of endogenous Cx43 via ACT1, and could point to important non-junctional functions for Cx43 when overexpressed. However, the details of this claim remain to be tested. Based on our results, we assert that modulating gap junction activity of endogenous Cx43 protein is an important aspect of these studies and our findings are consistent with a tumor suppressive role for Cx43. Moreover, these findings indicate that treating breast cancer cells with ACT1 is therapeutically beneficial due to its ability to impair breast cancer cell proliferation.

Interestingly, we observe differential effects of ACT1 on proliferation between non-transformed MCF10A cells and the MCF7 and MDA MB 231 breast cancer cell lines. While this finding is a positive outcome for the therapeutic use of ACT1, since normal cell function will likely not be affected by ACT1 usage, this indicates that differential regulation or activity of Cx43 could exist in different cell types. Indeed, we found that Cx43 expression, localization, and gap junction activity was not altered in response to ACT1 in MCF10A cells. Additionally, we found that BT474 cells responded differently to ACT1. In the BT474 cell line, cell number was not affected by ACT1. Our data suggests, at least in part, this difference in activity could be explained by a variation in Cx43 and ZO-1 levels in the different cell lines. We report that Cx43 levels are reduced and ZO-1 levels are increased in BT474 cells, which would likely affect Cx43 activity since ZO-1 inhibits the gap junction activity of Cx43 [33,34]. Interestingly, we also found that ACT1 induced apoptosis in BT474 cells but not in MCF10A or MCF7 cells. These additional findings suggest that the MCF7 and BT474 cells have different levels of sensitivity and/or response to ACT1.

Interestingly, Cx43 levels were low in MCF10A cells but were somewhat higher in the breast cancer cell lines we evaluated. This raises the question of whether total Cx43 levels are an appropriate analysis for Cx43’s role in breast cancer, or if evaluating localization and/or activity is more germane to establishing Cx43’s role. Moreover, our observation that ZO-1 levels vary between cell types should be taken into consideration when assessing Cx43 function in breast cancers, as ZO-1 negatively regulates Cx43 gap junctional activity. Perhaps a better approach would be to evaluate both Cx43 and ZO-1 in concert with one another for a more complete picture. We additionally evaluated the expression levels of two other connexin proteins, Cx26 and Cx46, which have been reported in breast cancer [13,49], and found that these proteins were more robustly expressed in all three breast cancer cells lines when compared to MCF10A cells (Additional file 1: Figure S6). However, the significance of this finding remains to be addressed.

Recent evidence showed that the gap junction restoring agent, PQ1, modulated Cx43 gap junction activity in a colon cancer model [32]. Although PQ1 alters Cx43 activity, it is not specific for Cx43 as it also alters Cx46 expression [32,50] and may induce off-target effects [51]. One potential concern with the activity of PQ1 is that it induces activation of AKT and the MAPKs ERK1/2 [32], which are tumor promoting factors. Our findings indicate that ACT1 treatment of MCF7 cells does not alter AKT or ERK1/2 activity. Therefore, while PQ1 shows therapeutic promise through targeting junctional connexins and modulating apoptosis, the concomitant increases in AKT and MAPK activity could eventually contribute to therapeutic resistance. In line with this theory, AKT and MAPK pathway alterations occur at high frequency in breast cancers that become therapeutically resistant [52]. Our results indicate that therapeutic application of ACT1 would not share this effect.

While expression studies in human breast cancer samples have shown that there is a loss of junctional Cx43 expression [17], previous findings have also indicated that total Cx43 levels can fluctuate with breast cancer stage [14-16,19]. One possibility is that impairing Cx43 from reaching gap junctions, whether it be by downregulation or mislocalization, is a primary adaptation for breast tumor cells. Indeed, some reports indicate that a Cx43 pseudogene exists in some cancers, which likely impairs Cx43 activity [53]. However, other studies provide evidence that non-junctional activities of Cx43 are likely important as well [36,40]. At least two non-junctional mechanisms have been proposed for Cx43, autophagy and apoptosis. Certainly, these processes have been shown to impact breast cancer progression and treatment [54]. Our current studies suggest that treatment of MCF7 cells with the Cx43-targeted drug, ACT1, does not impact autophagy, a result that is consistent with previous findings that Cx43 inhibits autophagy in a gap junction independent manner [40]. Previous findings indicate that exogenous expression of Cx43 reduces the expression of Bcl-2, an apoptosis inhibitory protein, and induces apoptosis in a gap-junction independent manner [36]. Since ACT1 specifically sequesters Cx43 to gap junctions, it is unlikely that ACT1 alters Bcl-2 expression status but this remains to be tested. Additional investigation into the relationship between Cx43 and these processes would be an important future endeavor.

Cx43 is regulated by the post-translational phosphorylation of serines (S364, S365, S325, S328, S330, S368, S279, S282, S262) in the C-terminal domain of Cx43 and depending on the site of phosphorylation, these processes contribute to conformational changes and the formation or disruption of gap junctions and gap junction intercellular communication [55-58]. Importantly, ACT1 has been shown to increase the phosphorylation of S368 in an injury-dependent manner [59]. Whether S368 phosphorylation is affected by ACT1 in cancer cells may be an important factor in mediating this bystander effect by creating a selective “distinct communication compartment” [57]. Because a number of these Cx43 phosphorylation sites are reported to regulate the localization of Cx43 independently of gap junction activity it is plausible that non-junctional roles of Cx43 are also directly regulated by phosphorylation events. This aspect of Cx43 signaling remains to be uncovered and further applied to understanding Cx43’s role in breast cancer.

In our present study we show combinatorial ACT1 treatment with tamoxifen and lapatinib enhanced the effectiveness of these agents in MCF7 and BT474 breast cancer cells, respectively. However, while ACT1 treatment alone was able to alter MCF7 proliferation, it did not appear to have a robust effect on BT474 cell number. In addition to the different expression levels of Cx43 and ZO-1 we report, this result may be linked to differences in Cx43 activity in ER-dependent versus HER2-dependent cells. In support of the possibility that differential effects could be observed in ER+ cells, previous studies have shown that Cx43 expression is regulated by estrogen and is inversely correlated with ER expression in human myometrial cultures and tissues [60,61]. Genome expression analysis suggests that high levels of Cx43 improves survival outcome of breast cancer patients with ER+ tumors but impairs survival outcome in ER- and HER2+ cohorts [62,63]. Consequently, the contribution of specific oncogenic drivers may have an effect on Cx43 expression or activity in breast cancer cells of different subtypes. Likewise, tamoxifen could influence Cx43 expression or activity. These disparities would presumably impact ACT1’s activity toward Cx43 differentially in each individual breast cancer cell subtype, similar to the results that we report here. We note that BT474 cells express ER, and certainly ER may be modulated by ACT1 in these cells, but the proliferative capacity of BT474 cells is largely dependent on HER2 as the primary oncogenic driver. While additional investigation is required to further elucidate ACT1’s impact on ER expression, this line of reasoning provides possible additional explanation for the differences in proliferation and apoptosis we observe due to ACT1 treatment alone in MCF7 cells compared with the response found in BT474 cells.

Cx43 has been investigated in a HER2 model where investigators used genetically engineered mouse models to express a mutant form of Cx43 containing a glutamine to a serine alteration at position 60 (G60S) that localizes to gap junctions but has impaired gap junction intercellular communication [28]. The researchers found that crossing G60S mutant mice with HER2-overexpressing mice in which tumorigenesis is induced by carcinogen (DMBA) exposure revealed unexpected findings where G60S mice had delayed tumor onset but increased metastasis [28]. Because of the use of DMBA as a carcinogen and the gap junction dependent “bystander” effect, we are left to question if the delayed tumor onset, despite loss of Cx43-gap junction activity, could be due to an inability for G60S animals to “see” the intercellular carcinogenic effects of DMBA. Further studies are required to clarify these findings. Regardless, our findings suggest that the combination of targeting Cx43 with ACT1 with targeted inhibitors, demonstrated here with tamoxifen and lapatinib, is beneficial in breast cancer.

Finally, we found that combining ACT1 and lapatinib reduced primary mammosphere formation in the BT474 model. Because mammosphere formation is typically associated with cancer stem cell activity and survival of cancer stem cells impacts cancer therapy as well as development of resistance to cancer therapy [39], it is enticing to speculate that modulation of Cx43 regulates cancer stem cell proliferation or survival. However, as we have only assessed primary mammosphere formation and not propagation or self-renewal, care must be taken when interpreting these findings. While our results presented in Figure 6 support our initial findings in 2D culture systems (Figure 5), additional studies are required in order to determine whether ACT1 alters proliferation or survival of tumor initiating cells or if it impacts tumor stem cell renewal.

## Conclusions

Our findings demonstrate that targeting the gap junctional distribution and activity of Cx43 using ACT1 is effective in breast cancer. Furthermore, ACT1 enhances the activity of the targeted therapies, tamoxifen and lapatinib, supporting the clinical potential of combinational strategies that include modulation of Cx43 by ACT1.

## Additional file

Additional file 1: Figure S1. Immunoblotting for total Cx43 protein levels in whole cell extracts from MCF7 cells treated with vehicle, R-pep, or ACT. **Figure S2.** (A) Immunoblotting for LC3B processing in MCF7 cells treated with vehicle, R-pep, or ACT, in the presence or absence of chloroquine (100 μM). (B) Immunoblotting for pAKT and pERK1/2 in whole cell extracts from MCF7 cells treated with vehicle, R-pep, or ACT. **Figure S3.** (A) MCF10A cells were treated with R-pep (200 μM), or ACT1 (200 μM) and assessed for Immunofluoresence staining and imaging of Cx43 (green). Wheat germ agglutinin (WGA) in red was used to stain cell membranes. (B) MCF10A cells were treated with R-pep (200 μM) or ACT1 (200 μM) and assessed for gap-FRAP. **Figure S4.** Connexin 43 activity and expression after ACT1 treatment in BT474 cells. BT474 cells were treated with vehicle, R-pep (200 μM), or ACT1 (200 μM) and assessed for (A) gap-FRAP. Dunnett’s Multiple Comparison Test was used to determine statistical significance *= p<0.05 vs Vehicle or R-Pep; ± SEM (B) Immunofluoresence staining and imaging of Cx43 (green) in BT474 cells treated with R-pep or ACT1. Wheat germ agglutinin (WGA) in red was used to stain cell membranes. **Figure S5.** p27 expression levels are not altered by ACT1. Whole cell extracts isolated from MCF10A, MCF7, and BT474 cells treated with R-pep (100 μM), or ACT1 (100 μM) were immunoblotted for p27. **Figure S6.** Differential expression of Cx26 and Cx46 in non-transformed breast epithelial cells and breast cancer cell lines. Whole cell extracts isolated from MCF10A, MCF7, BT474, and MDA MB 231 cells were immunoblotted for Cx26 and Cx46.

## Abbreviations

Cx: Connexin
Cx43: Connexin 43
ACT1: α–connexin carboxyl-terminal
R-pep: Reverse peptide
HER2: Human Epidermal Growth Factor Receptor 2
ER: Estrogen Receptor
kDa: kilodalton
FRAP: Fluorescence Recovery After Photobleaching
AKT: Protein Kinase B
MAPK: Mitogen Activated Protein Kinase
ERK: Extracellular signal-Regulated Kinase
LC3B: Light Chain 3B
Bcl-2: B-cell lymphoma-2

## References

[1] Grek CL, Rhett JM, Ghatnekar GS (2014). Cardiac to cancer: connecting connexins to clinical opportunity. FEBS Lett.

[2] Kumar NM, Gilula NB (1996). The gap junction communication channel. Cell.

[3] Makowski L, Caspar DL, Phillips WC, Goodenough DA (1977). Gap junction structures. II. Analysis of the x-ray diffraction data. J Cell Biol.

[4] Naus CC, Laird DW (2010). Implications and challenges of connexin connections to cancer. Nat Rev Cancer.

[5] Monaghan P, Moss D (1996). Connexin expression and gap junctions in the mammary gland. Cell Biol Int.

[6] Plante I, Laird DW (2008). Decreased levels of connexin43 result in impaired development of the mammary gland in a mouse model of oculodentodigital dysplasia. Dev Biol.

[7] Plante I, Stewart MK, Laird DW (2011). Evaluation of mammary gland development and function in mouse models. J Vis Exp.

[8] Plante I, Wallis A, Shao Q, Laird DW (2010). Milk secretion and ejection are impaired in the mammary gland of mice harboring a Cx43 mutant while expression and localization of tight and adherens junction proteins remain unchanged. Biol Reprod.

[9] Stewart MK, Gong XQ, Barr KJ, Bai D, Fishman GI, Laird DW (2013). The severity of mammary gland developmental defects is linked to the overall functional status of Cx43 as revealed by genetically modified mice. Biochem J.

[10] Yamanaka I, Kuraoka A, Inai T, Ishibashi T, Shibata Y (1997). Changes in the phosphorylation states of connexin43 in myoepithelial cells of lactating rat mammary glands. Eur J Cell Biol.

[11] Elzarrad MK, Haroon A, Willecke K, Dobrowolski R, Gillespie MN, Al-Mehdi AB (2008). Connexin-43 upregulation in micrometastases and tumor vasculature and its role in tumor cell attachment to pulmonary endothelium. BMC Med.

[12] Gould VE, Mosquera JM, Leykauf K, Gattuso P, Durst M, Alonso A (2005). The phosphorylated form of connexin43 is up-regulated in breast hyperplasias and carcinomas and in their neoformed capillaries. Hum Pathol.

[13] Jamieson S, Going JJ, D’Arcy R, George WD (1998). Expression of gap junction proteins connexin 26 and connexin 43 in normal human breast and in breast tumours. J Pathol.

[14] Kanczuga-Koda L, Sulkowska M, Koda M, Reszec J, Famulski W, Baltaziak M (2003). Expression of connexin 43 in breast cancer in comparison with mammary dysplasia and the normal mammary gland. Folia Morphol.

[15] Kanczuga-Koda L, Sulkowski S, Lenczewski A, Koda M, Wincewicz A, Baltaziak M (2006). Increased expression of connexins 26 and 43 in lymph node metastases of breast cancer. J Clin Pathol.

[16] Kanczuga-Koda L, Sulkowski S, Tomaszewski J, Koda M, Sulkowska M, Przystupa W (2005). Connexins 26 and 43 correlate with Bak, but not with Bcl-2 protein in breast cancer. Oncol Rep.

[17] Laird DW, Fistouris P, Batist G, Alpert L, Huynh HT, Carystinos GD (1999). Deficiency of connexin43 gap junctions is an independent marker for breast tumors. Cancer Res.

[18] Li Z, Zhou Z, Welch DR, Donahue HJ (2008). Expressing connexin 43 in breast cancer cells reduces their metastasis to lungs. Clin Exp Metastasis.

[19] Kanczuga-Koda L, Sulkowska M, Koda M, Rutkowski R, Sulkowski S (2007). Increased expression of gap junction protein–connexin 32 in lymph node metastases of human ductal breast cancer. Folia Histoche Cytobiol/Pol Acad Sci Pol Histochem Cytochem Soc.

[20] Locke D (1998). Gap junctions in normal and neoplastic mammary gland. J Pathol.

[21] Laird DW (2006). Life cycle of connexins in health and disease. Biochem J.

[22] Langlois S, Cowan KN, Shao Q, Cowan BJ, Laird DW (2010). The tumor-suppressive function of Connexin43 in keratinocytes is mediated in part via interaction with caveolin-1. Cancer Res.

[23] Ito A, Watabe K, Koma Y, Kitamura Y (2002). An attempt to isolate genes responsible for spontaneous and experimental metastasis in the mouse model. Histol Histopathol.

[24] Chao Y, Wu Q, Acquafondata M, Dhir R, Wells A (2012). Partial mesenchymal to epithelial reverting transition in breast and prostate cancer metastases. Cancer Microenviron.

[25] Solan JL, Hingorani SR, Lampe PD (2012). Changes in connexin43 expression and localization during pancreatic cancer progression. J Membr Biol.

[26] McLachlan E, Shao Q, Wang HL, Langlois S, Laird DW (2006). Connexins act as tumor suppressors in three-dimensional mammary cell organoids by regulating differentiation and angiogenesis. Cancer Res.

[27] Momiyama M, Omori Y, Ishizaki Y, Nishikawa Y, Tokairin T, Ogawa J (2003). Connexin26-mediated gap junctional communication reverses the malignant phenotype of MCF-7 breast cancer cells. Cancer Sci.

[28] Plante I, Stewart MK, Barr K, Allan AL, Laird DW (2011). Cx43 suppresses mammary tumor metastasis to the lung in a Cx43 mutant mouse model of human disease. Oncogene.

[29] Pollmann MA, Shao Q, Laird DW, Sandig M (2005). Connexin 43 mediated gap junctional communication enhances breast tumor cell diapedesis in culture. Breast Cancer Res.

[30] Qin H, Shao Q, Curtis H, Galipeau J, Belliveau DJ, Wang T (2002). Retroviral delivery of connexin genes to human breast tumor cells inhibits in vivo tumor growth by a mechanism that is independent of significant gap junctional intercellular communication. J Biol Chem.

[31] Talhouk RS, Fares MB, Rahme GJ, Hariri HH, Rayess T, Dbouk HA (2013). Context dependent reversion of tumor phenotype by connexin-43 expression in MDA-MB231 cells and MCF-7 cells: role of beta-catenin/connexin43 association. Exp Cell Res.

[32] Bigelow K, Nguyen TA (2014). Increase of gap junction activities in SW480 human colorectal cancer cells. BMC Cancer.

[33] Rhett JM, Jourdan J, Gourdie RG (2011). Connexin 43 connexon to gap junction transition is regulated by zonula occludens-1. Mol Biol Cell.

[34] Hunter AW, Barker RJ, Zhu C, Gourdie RG (2005). Zonula occludens-1 alters connexin43 gap junction size and organization by influencing channel accretion. Mol Biol Cell.

[35] Spray DC, Hanstein R, Lopez-Quintero SV, Stout RF, Suadicani SO, Thi MM (2013). Gap junctions and Bystander Effects: Good Samaritans and executioners. Wiley Interdiscipl Rev Membrane Transp Signal.

[36] Huang RP, Hossain MZ, Huang R, Gano J, Fan Y, Boynton AL (2001). Connexin 43 (cx43) enhances chemotherapy-induced apoptosis in human glioblastoma cells. Int J Cancer.

[37] Carystinos GD, Alaoui-Jamali MA, Phipps J, Yen L, Batist G (2001). Upregulation of gap junctional intercellular communication and connexin 43 expression by cyclic-AMP and all-trans-retinoic acid is associated with glutathione depletion and chemosensitivity in neuroblastoma cells. Cancer Chemother Pharmacol.

[38] Munoz JL, Rodriguez-Cruz V, Greco SJ, Ramkissoon SH, Ligon KL, Rameshwar P (2014). Temozolomide resistance in glioblastoma cells occurs partly through epidermal growth factor receptor-mediated induction of connexin 43. Cell Death Dis.

[39] Shaw FL, Harrison H, Spence K, Ablett MP, Simoes BM, Farnie G (2012). A detailed mammosphere assay protocol for the quantification of breast stem cell activity. J Mammary Gland Biol Neoplasia.

[40] Bejarano E, Yuste A, Patel B, Stout RF, Spray DC, Cuervo AM (2014). Connexins modulate autophagosome biogenesis. Nat Cell Biol.

[41] Dunn CA, Lampe PD (2014). Injury-triggered Akt phosphorylation of Cx43: a ZO-1-driven molecular switch that regulates gap junction size. J Cell Sci.

[42] Dunn CA, Su V, Lau AF, Lampe PD (2012). Activation of Akt, not connexin 43 protein ubiquitination, regulates gap junction stability. J Biol Chem.

[43] Warn-Cramer BJ, Cottrell GT, Burt JM, Lau AF (1998). Regulation of connexin-43 gap junctional intercellular communication by mitogen-activated protein kinase. J Biol Chem.

[44] McLachlan E, Shao Q, Laird DW (2007). Connexins and gap junctions in mammary gland development and breast cancer progression. J Membr Biol.

[45] Sanchez-Alvarez R, Paino T, Herrero-Gonzalez S, Medina JM, Tabernero A (2006). Tolbutamide reduces glioma cell proliferation by increasing connexin43, which promotes the up-regulation of p21 and p27 and subsequent changes in retinoblastoma phosphorylation. Glia.

[46] Zhang YW, Morita I, Ikeda M, Ma KW, Murota S (2001). Connexin43 suppresses proliferation of osteosarcoma U2OS cells through post-transcriptional regulation of p27. Oncogene.

[47] Zhang YW, Nakayama K, Nakayama K, Morita I (2003). A novel route for connexin 43 to inhibit cell proliferation: negative regulation of S-phase kinase-associated protein (Skp 2). Cancer Res.

[48] Rhett JM, Ongstad EL, Jourdan J, Gourdie RG (2012). Cx43 associates with Na(v)1.5 in the cardiomyocyte perinexus. J Membr Biol.

[49] Teleki I, Krenacs T, Szasz MA, Kulka J, Wichmann B, Leo C (2013). The potential prognostic value of connexin 26 and 46 expression in neoadjuvant-treated breast cancer. BMC ancer.

[50] Ding Y, Nguyen TA (2012). Gap Junction Enhancer Potentiates Cytotoxicity of Cisplatin in Breast Cancer Cells. J Cancer Sci Ther.

[51] Ding Y, Nguyen TA (2013). PQ1, a quinoline derivative, induces apoptosis in T47D breast cancer cells through activation of caspase-8 and caspase-9. Apoptosis.

[52] Britten CD (2013). PI3K and MEK inhibitor combinations: examining the evidence in selected tumor types. Cancer Chemother Pharmacol.

[53] Kandouz M, Bier A, Carystinos GD, Alaoui-Jamali MA, Batist G (2004). Connexin43 pseudogene is expressed in tumor cells and inhibits growth. Oncogene.

[54] Maiuri MC, Zalckvar E, Kimchi A, Kroemer G (2007). Self-eating and self-killing: crosstalk between autophagy and apoptosis. Nat Rev Mol Cell Biol.

[55] Musil LS, Goodenough DA (1991). Biochemical analysis of connexin43 intracellular transport, phosphorylation, and assembly into gap junctional plaques. J Cell Biol.

[56] Richards TS, Dunn CA, Carter WG, Usui ML, Olerud JE, Lampe PD (2004). Protein kinase C spatially and temporally regulates gap junctional communication during human wound repair via phosphorylation of connexin43 on serine368. J Cell Biol.

[57] Solan JL, Lampe PD (2009). Connexin43 phosphorylation: structural changes and biological effects. Biochem J.

[58] Solan JL, Lampe PD (2007). Key connexin 43 phosphorylation events regulate the gap junction life cycle. J Membr Biol.

[59] Palatinus JA, Rhett JM, Gourdie RG (2011). Enhanced PKCepsilon mediated phosphorylation of connexin43 at serine 368 by a carboxyl-terminal mimetic peptide is dependent on injury. Channels.

[60] Yu W, Dahl G, Werner R (1994). The connexin43 gene is responsive to oestrogen. Proc Biol Sci/Royal Soc.

[61] Andersen J (2000). Comparing regulation of the connexin43 gene by estrogen in uterine leiomyoma and pregnancy myometrium. Environ Health Perspect.

[62] Teleki I, Szasz AM, Maros ME, Gyorffy B, Kulka J, Meggyeshazi N (2014). Correlations of differentially expressed gap junction connexins Cx26, Cx30, Cx32, Cx43 and Cx46 with breast cancer progression and prognosis. PLoS One.

[63] Gyorffy B, Lanczky A, Eklund AC, Denkert C, Budczies J, Li Q (2010). An online survival analysis tool to rapidly assess the effect of 22,277 genes on breast cancer prognosis using microarray data of 1,809 patients. Breast Cancer Res Treat.

