# Supplementary material for: Targeting connexin 43 with α–connexin carboxyl-terminal (ACT1) peptide enhances the activity of the targeted inhibitors, tamoxifen and lapatinib, in breast cancer: clinical implication for ACT1
Source: BMC Cancer. 2015 Apr 3;15:296. doi: 10.1186/s12885-015-1229-6 (PMC4407347; doi:10.1186/s12885-015-1229-6)

Supplementary Figure 1

Immunoblotting for total Cx43 protein levels in whole cell extracts from MCF7 cells treated with vehicle, R-pep, or ACT.

Supplementary Figure 2

(A) Immunoblotting for LC3B processing in MCF7 cells treated with vehicle, R-pep, or ACT, in the presence or absence of chloroquine (100 μM). (B) Immunoblotting for pAKT and pERK1/2 in whole cell extracts from MCF7 cells treated with vehicle, R-pep, or ACT.

Supplementary Figure 3.

(A) MCF10A cells were treated with R-pep (200 μM), or ACT1 (200 μM) and assessed for Immunofluoresence staining and imaging of Cx43 (green). Wheat germ agglutinin (WGA) in red was used to stain cell membranes. (B) MCF10A cells were treated with R-pep (200 μM) or ACT1 (200 μM) and assessed for gap-FRAP.

Supplementary Figure 4.

Connexin 43 activity and expression after ACT1 treatment in BT474 cells. BT474 cells were treated with vehicle, R-pep (200 μM), or ACT1 (200 μM) and assessed for (A) gap-FRAP. Dunnett's Multiple Comparison Test was used to determine statistical significance * = $p<0.05$ vs Vehicle or R-Pep; ± SEM (B) Immunofluoresence staining and imaging of Cx43 (green) in BT474 cells treated with R-pep or ACT1. Wheat germ agglutinin (WGA) in red was used to stain cell membranes.

Supplementary Figure 5.

p27 expression levels are not altered by ACT1. Whole cell extracts isolated from MCF10A, MCF7, and BT474 cells treated with R-pep (100 μM), or ACT1 (100 μM) were immunoblotted for p27.

Supplementary Figure 6.

Differential expression of Cx26 and Cx46 in non-transformed breast epithelial cells and breast cancer cell lines. Whole cell extracts isolated from MCF10A, MCF7, BT474, and MDA MB 231 cells were immunoblotted for Cx26 and Cx46.

Supplementary Figure 1

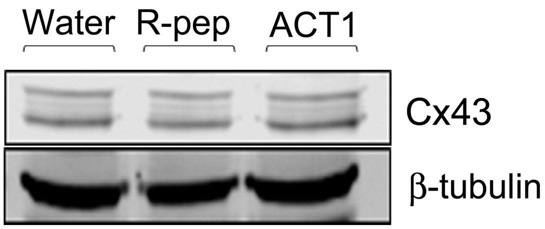

Supplmentary Figure 2

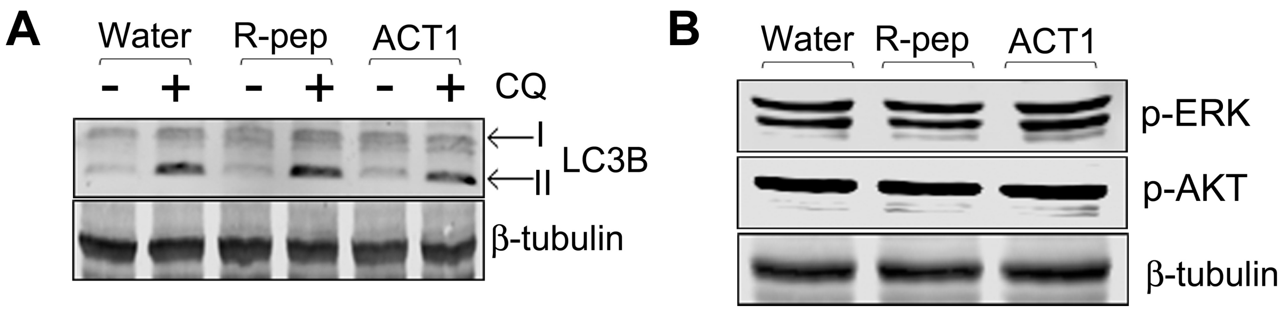

Supplementary Figure 3

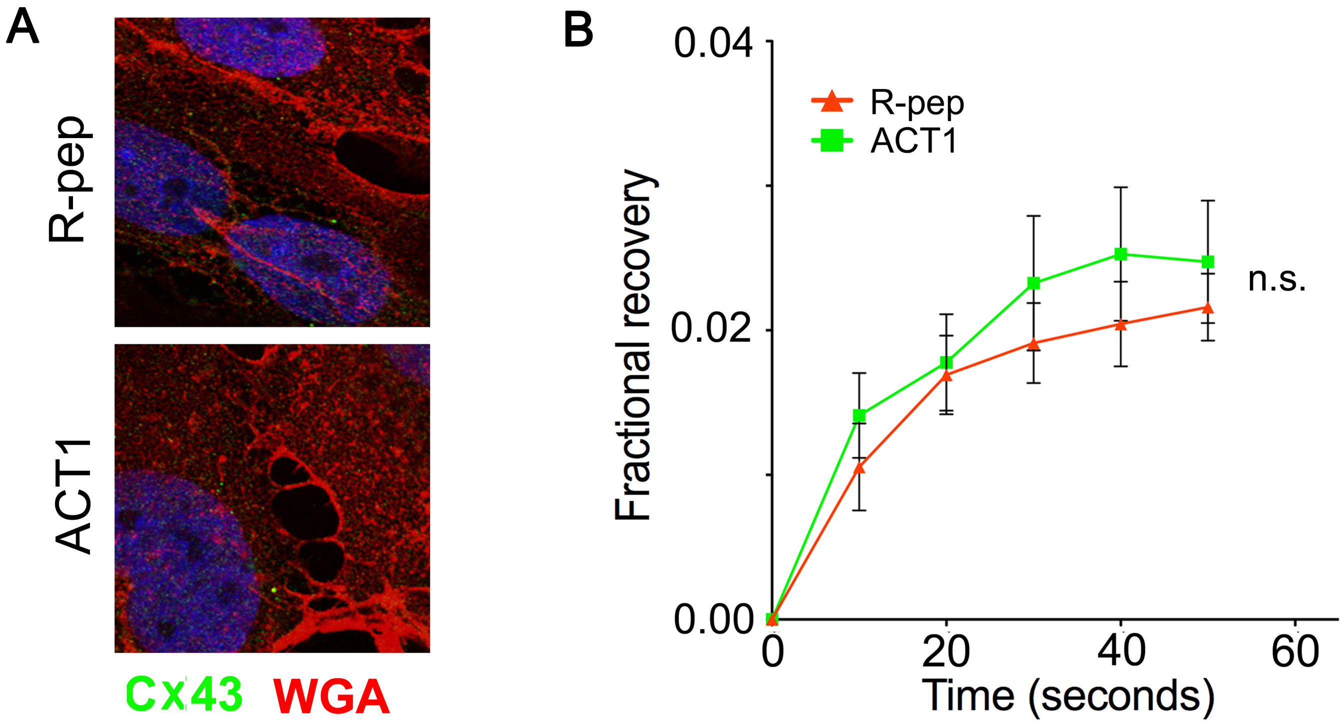

Supplementary Figure 4

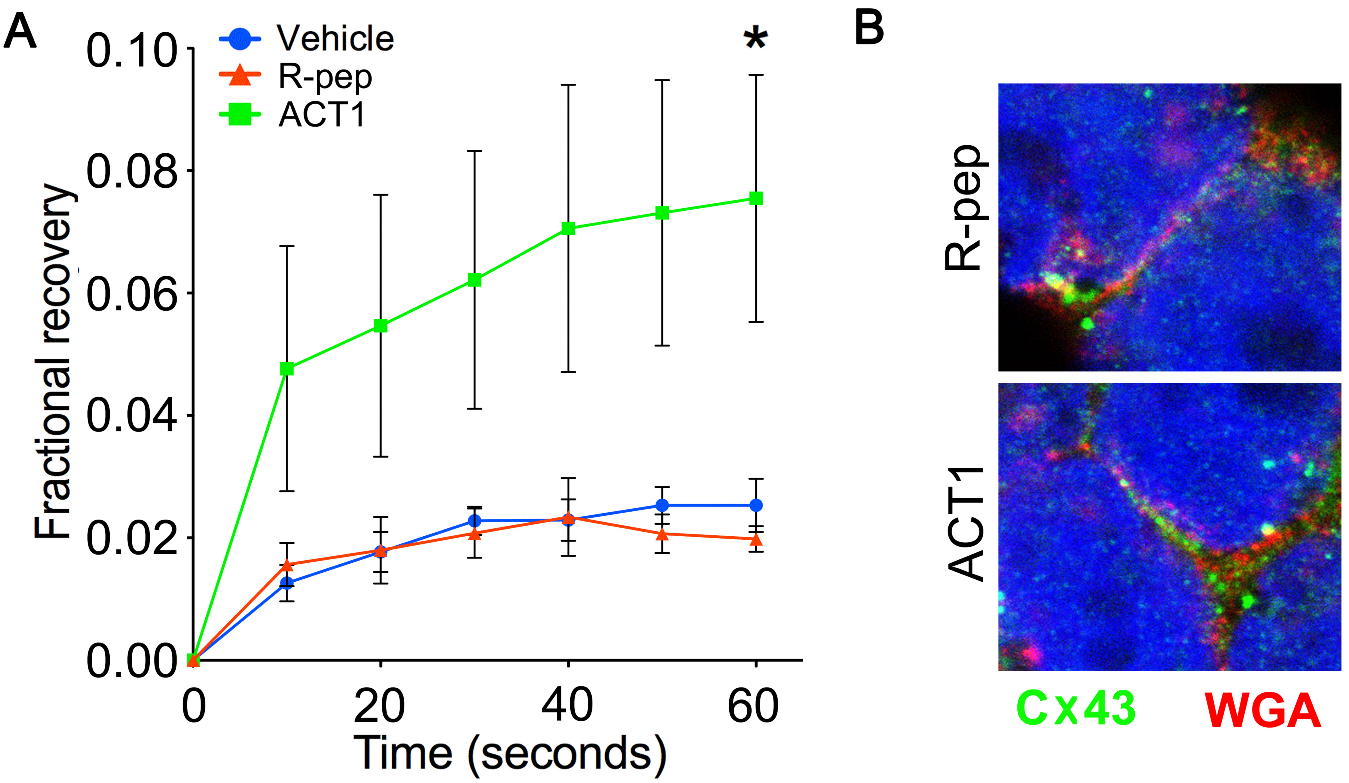

Supplementary Figure 5

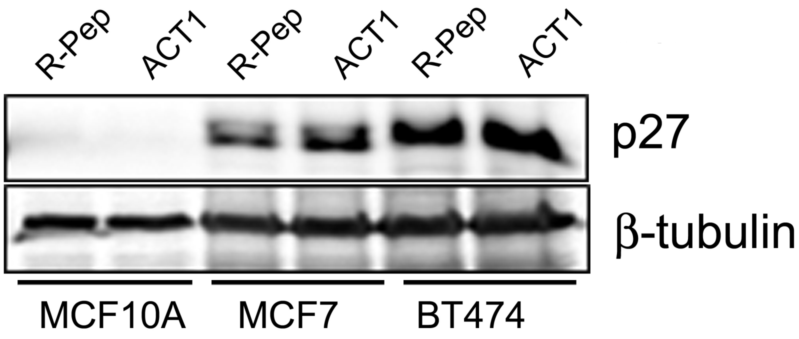

Supplementary Figure 6

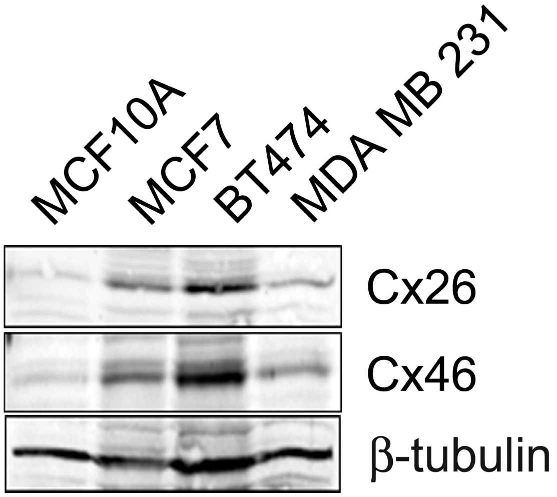

Supplement: Additional file 1: Figure S1. — Immunoblotting for total Cx43 protein levels in whole cell extracts from MCF7 cells treated with vehicle, R-pep, or ACT. Figure S2. (A) Immunoblotting for LC3B processing in MCF7 cells treated with vehicle, R-pep, or ACT, in the presence or absence of chloroquine (100 μM). (B) Immunoblotting for pAKT and pERK1/2 in whole cell extracts from MCF7 cells treated with vehicle, R-pep, or ACT. Figure S3. (A) MCF10A cells were treated with R-pep (200 μM), or ACT1 (200 μM) and assessed for Immunofluoresence staining and imaging of Cx43 (green). Wheat germ agglutinin (WGA) in red was used to stain cell membranes. (B) MCF10A cells were treated with R-pep (200 μM) or ACT1 (200 μM) and assessed for gap-FRAP. Figure S4. Connexin 43 activity and expression after ACT1 treatment in BT474 cells. BT474 cells were treated with vehicle, R-pep (200 μM), or ACT1 (200 μM) and assessed for (A) gap-FRAP. Dunnett’s Multiple Comparison Test was used to determine statistical significance *= p<0.05 vs Vehicle or R-Pep; ± SEM (B) Immunofluoresence staining and imaging of Cx43 (green) in BT474 cells treated with R-pep or ACT1. Wheat germ agglutinin (WGA) in red was used to stain cell membranes. Figure S5. p27 expression levels are not altered by ACT1. Whole cell extracts isolated from MCF10A, MCF7, and BT474 cells treated with R-pep (100 μM), or ACT1 (100 μM) were immunoblotted for p27. Figure S6. Differential expression of Cx26 and Cx46 in non-transformed breast epithelial cells and breast cancer cell lines. Whole cell extracts isolated from MCF10A, MCF7, BT474, and MDA MB 231 cells were immunoblotted for Cx26 and Cx46. [file 12885_2015_1229_MOESM1_ESM.pdf]
